# Understanding Patient Experiences, Opinions, and Actions Taken After Viewing Their Own Radiology Images Online: Web-Based Survey

**DOI:** 10.2196/29496

**Published:** 2022-04-25

**Authors:** Evan C Norris, Ciarra Halaska, Peter B Sachs, Chen-Tan Lin, Kate Sanfilippo, Justin M Honce

**Affiliations:** 1 Department of Radiology University of Colorado Anschutz Medical Campus Aurora, CO United States; 2 UCHealth Aurora, CO United States; 3 Department of Medicine University of Colorado Anschutz Medical Campus Aurora, CO United States

**Keywords:** personal health records, patient-accessible electronic health records, online radiology image viewing, imaging informatics, patient portals, electronic health records, digital health, mobile phone

## Abstract

**Background:**

The ability for patients to directly view their radiology images through secure electronic portals is rare in the American health care system. We previously surveyed patients within our health system and found that a large majority wanted to view their own radiology images online, and we have since implemented this new feature.

**Objective:**

We aim to understand patient experiences, opinions, and actions taken after viewing their own radiology images online.

**Methods:**

We emailed a web-based survey to patients who recently viewed their radiology images via our electronic patient portal.

**Results:**

We sent 1825 surveys to patients and received 299 responses (response rate 16.4%). Patients reported a favorable experience (258/299, 86.3% agree) viewing their radiology images online. Patients found value in reading their radiology reports (288/299, 96.3% agree) and viewing their images (267/299, 89.3% agree). Overall, patients felt that accessing and viewing their radiology images online increased their understanding of their medical condition (258/299, 82.9%), made them feel more in control and reassured (237/299, 79.2% and 220/299, 73.6%, respectively), and increased levels of trust (214/299, 71.6%). Only 6.4% (19/299) of the patients indicated concerns with finding errors, 6.4% (19/299) felt that viewing their images online made them worry more, and 7% (21/299) felt confused when viewing their images online. Of patients who viewed their images online, 45.2% (135/299) took no action with their images, 32.8% (98/299) saved a copy for their records, 25.4% (76/299) shared them with their doctor, and 14.7% (44/299) shared them with another doctor for a second opinion. A total of 9 patients (3%) shared their radiology images on Facebook, Instagram, or both, primarily to inform family and friends. Approximately 10.4% (31/299) of the patients had questions about their radiology images after viewing them online, with the majority (20/31, 65%) seeking out a doctor, and far fewer (5/31, 16%) choosing to ask a family member about their images. Finally, respondents viewed their images online using 1 or more devices, including computers, smartphones, tablets, or a combination of these devices. Approximately 26.7% (103/385) of the responses noted technical difficulties, with the highest incidence rate occurring with smartphones.

**Conclusions:**

We report the first known survey results from patients who viewed their own radiology images through a web-based portal. Patients reported high levels of satisfaction and increased levels of trust, autonomy, reassurance, and medical understanding. Only a small minority of patients expressed anxiety or confusion. We suggest that patient access to radiology images, such as patient access to radiology reports, is highly desired by patients and is operationally practical. Other health care institutions should consider offering patients access to their radiology images online in the pursuit of information transparency.

## Introduction

Hospital systems and providers (health care professionals) around the country have increasingly implemented web-based patient portals, which allow patients to directly access portions of their electronic health record data, including laboratory test results, radiology reports, and pathology reports [1-3]. This new and unprecedented transparency between patients and their health information has been spurred forward by the information-blocking provision of the 21st Century Cures Act, which requires patients to be granted immediate access to clinical information entered into the electronic health record, including radiology reports [4]. The expansion of patient health information availability and obtainability has led to improvements in communication between patients and their providers as well as increased patient-centered care [4-6]. Until recently, however, patients were only able to read their radiology reports and could not view their personal radiographic images themselves. Health systems that have integrated image viewing portals into the patient accessible electronic record have documented up to >7-fold increases in the numbers of patients viewing their images, indicating a strong patient interest in the ability to view images [7]. Such interest has been corroborated by surveys demonstrating patient interest in viewing their radiology images and patient perception that there are potential benefits from doing so [8-10], but they have never before been surveyed about their experiences after viewing their own images though a web-based patient portal.

In August 2018, UCHealth launched direct patient viewing of personal radiology images through its My Health Connection (MHC) web-based portal. Prior to the release of this feature, we conducted an independent preintervention survey aimed at better understanding patient attitudes toward viewing their radiology images online. A majority of the surveyed patients felt that the increased transparency far outweighed the associated risks [10]. Following the successful launch of online patient radiology image viewing at UCHealth through the MHC patient portal, we asked patients about their experiences interacting with this new feature. In this report, we present the results of the first known survey of patients who have viewed their own radiology images online.

## Methods

### Imaging Viewing Implementation

**Figure 1:**
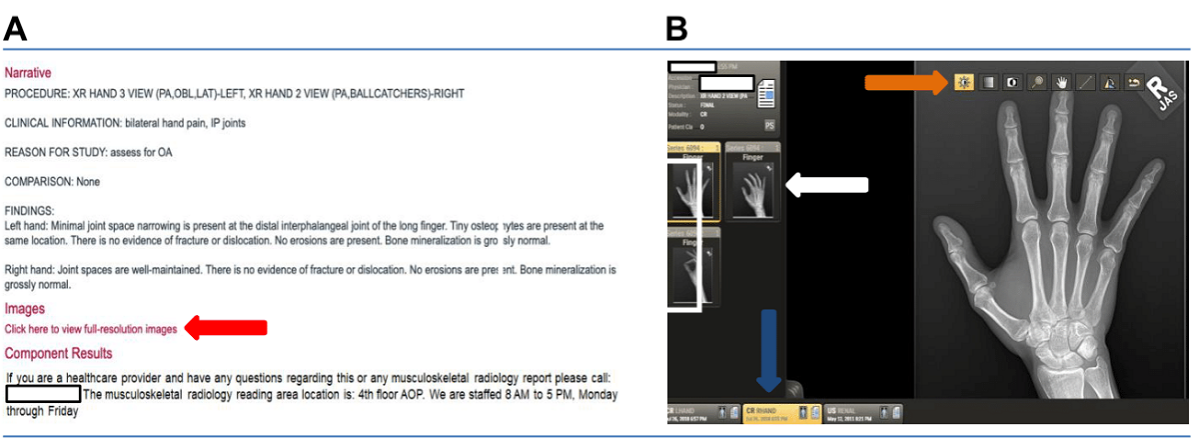
(A) Shows a sample musculoskeletal radiology report accessible to the patient via the My Health Connection web-based portal with a clickable link to view the associated images (red arrow). (B) Shows the imaging viewer with tabs for navigating all available images (white arrow) and studies (blue arrow), as well as modifying tool buttons (orange arrow) that provide patients with the ability to interact with their images. AOP: Anschutz Outpatient Pavilion; CR: conventional radiography; IP: interphalangeal; LAT: lateral; OA: osteoarthritis: OBL: oblique; PA: posterior-anterior; US: ultrasound; XR: x-ray.

Patients are able to view their images using our MHC patient portal. The MHC patient portal is available for patients to access both on desktop computers using a browser, as well as through tablets and mobile devices using a dedicated application. Upon viewing their report, they are able to click a link to load their images into a viewer originally designed for providers to view images remotely. They can then interact with these images via a series of tools/buttons on touch screen devices and mouse/keyboard on desktop/laptop computers as shown in Figure 1. All radiology imaging modalities are available for review through the portal, including radiography, computed tomography, magnetic resonance, ultrasound, fluoroscopy, and other images obtained during interventional procedures. Images are available immediately as soon as they are uploaded to our radiology picture and archiving communication provider and are not held pending review and released by the care team/doctor.

### Survey Design and Ethics Approval

This postintervention survey was designed to evaluate patient attitudes about viewing their own radiology images online, and a copy of the full survey is available in Multimedia Appendix 1. This survey had the support of the Chief Medical Officer of our health system and was reviewed by the Colorado Multiple Institutional Review Board (protocol #20-2593) who determined in an expedited review that the survey met ethics clearance guidelines, was deemed “quality improvement,” and did not require full review. The survey instrument can be found in Multimedia Appendix 1.

### Target Population

We included patients who had undergone radiology imaging and had viewed their images online via the MHC portal. The sample includes patients who underwent imaging both as inpatients and as outpatients.

### Recruitment

The surveyed patients were identified by querying our radiology picture and archiving communication provider for the most recent patients who viewed their radiology images within the web-based MHC portal over a 5-day period at the time of our study. There were 1998 patients from this list who could be matched to email addresses in our electronic health record. These patients were emailed a link to the survey. The initial recruitment email garnered 185 responses and was followed by 1 reminder notification 2 days later, after which an additional 114 responses were received.

### Statistical Analysis

Survey questions included both open- and closed-ended responses. After reading through all responses, open-ended responses were coded manually, sorting each response into a bucket. Buckets were developed by determining which themes came up most frequently. To ensure that we did not lose any information or make assumptions over implied importance, 1 response could be coded into multiple thematic buckets if multiple themes were present in the comment. For example, “I had to try to bring up the images multiple times before I could successfully bring up the images. It was also hard to navigate between images” was coded as both “Navigation issues” and “Required multiple refreshes/attempts to work.” Closed-ended responses were rated on a Likert-like scale. Likert scores were employed to report the survey results as percentages and were reflected as Top 2 Box scores aggregating scores of 4 or 5 from the 5-point scale. All statistical analyses were performed using Q Research Software Version 5.4.5.0 (Displayr).

## Results

### Target Population

Of the 1998 recruitment emails sent, 173 emails were returned to the sender and 1825 were successfully delivered. We received 299 complete responses, yielding a response rate of 16.4%. This response rate is within the expected range based on previous work completed in our health system. Approximately 69.2% (207/299) were female, 28.7% (86/299) were male, 1.3% (4/299) preferred not to answer, and 0.7% (2/299) identified with another gender. This gender demographic breakdown is in line with what is to be expected from an unweighted convenience sample, and as seen in Table 1, compares favorably with the percentage of patients in our institution who have undergone radiologic imaging. The majority of the respondents were aged 55 years or older (175/299, 58.5%). Regarding education levels, 28.4% (85/299) of the respondents held a bachelor’s degree, 23.1% (69/299) held a master’s degree, 14.8% (44/299) had some college, and 1% (3/299) did not complete high school. The majority of patients (271/299, 90.6%) had their radiology images taken as part of a clinic or outpatient radiology appointment, while far fewer were taken during an inpatient admission (18/299, 6%).

**Table 1:** Demographics of the patients.

Characteristic		Study population (N=299), n (%)	UCHealth imaging studies (N=650,843), n (%)
**Age (years)**			
	18-24	4 (1.3)	36,236 (5.6)
	25-34	31 (10.4)	79,274 (12.2)
	35-44	40 (13.3)	88,348 (13.6)
	45-54	49 (16.4)	95,413 (14.7)
	55-64	81 (27.1)	127,049 (19.5)
	65+	94 (31.4)	224,523 (34.5)
Female sex^a^		207 (69.2)	178,000 (64)

^a^Sex is reported for UCHealth data, as gender identity data were incomplete. The total number of imaging studies in female UCHealth patients was 278,000.

### Survey Results

When patients were asked on the postimplementation survey on a scale of 1 to 5 (1=poor, 5=excellent) about their overall experience viewing their radiology images in MHC, 86.3% (258/299) rated it favorably by the Top 2 Box score. Patients were asked to rate the value of viewing their radiology report and images online on a scale of 1 to 5 (1=not at all valuable, 5=extremely valuable), with the results shown in Table 2.

**Table 2:** Patient responses to the question, “Please rate the value of viewing each of the following within your web-based patient portal” (N=299).

Response	Agreement with statement, scored on a 5-point scale		
	Not valuable (bottom 2), n (%)	Neutral (middle 3), n (%)	Valuable (top 2), n (%)
Report	3 (0.7)	5 (1.7)	288 (96.3)
Images	8 (2.7)	22 (7.4)	267 (89.3)

Of the 299 respondents, 96.3% (288/299) of the patients rated viewing their radiology REPORT as valuable by the Top 2 Box score. Simultaneously, 89.3% (267/299) of the respondents rated viewing their IMAGES as valuable by Top 2 Box score. Surveyed patients rated their level of agreement with various statements regarding online viewing of their images as shown in Table 3. Approximately 82.9% (248/299) of the patients agreed that viewing radiology images would help “better understand my medical condition,” 79.2% (237/299) agreed that viewing their images made them “feel more in control” of their health care, 63.2% (189/299) agreed that viewing their radiology images online allowed them to “better follow their doctor’s recommendations,” and 73.6% (220/299) “felt reassured” that their doctor was doing the right thing. Moreover, 71.6% (214/299) of the patients agreed that viewing images online increased levels of trust. Lastly, 6.4% (19/299) of the patients indicated concerns with finding errors, 6.4% (19/299) felt that viewing their images online made them worried, and 7% (21/299) felt confused when viewing their images online. A total of 174 patients (58.2%) had questions about their radiology images after viewing them; 137 (45.8%) patients discussed their questions with their referring doctor, 14 (4.7%) patients discussed their questions with the radiologist, 23 (7.7%) patients had questions but did not ask anyone, and 30 (10%) patients asked someone else but did not specify who. We asked patients how they used their web-based images and with whom they wanted to discuss their images. As summarized in Table 4, 25.4% (76/299) reported sharing their images with their doctor and 14.7% (44/299) shared them with other doctors for a potential second opinion. Approximately 32.8% (98/299) saved a copy for their records, and 3% (9/299) shared them on social media.

**Table 3:** Distribution of agreement with statements regarding online viewing of images (N=299).

Statement: Viewing my radiology images online caused me to…	Agreement with statement, scored on a 5-point scale		
	Disagree (bottom 2), n (%)	Neutral (middle 3), n (%)	Agree (top 2), n (%)
...better understand my medical condition.	19 (6.4)	32 (10.7)	248 (82.9)
...feel more in control.	10 (3.3)	52 (17.4)	237 (79.3)
...feel reassured.	19 (6.4)	60 (20.1)	220 (73.6)
...trust my doctors more.	18 (6)	67 (22.4)	214 (71.6)
...better follow recommendations.	28 (9.4)	82 (27.4)	189 (63.2)
...feel confused/have a lot of questions.	229 (76.6)	49 (16.4)	21 (7)
...worry more.	250 (83.6)	30 (10)	19 (6.4)
...find errors in my radiology reports.	239 (79.9)	41 (13.7)	19 (6.4)

**Table 4:** Patient responses to the question, “Which of the following have you done with your radiology images? Select all that apply” (N=299).

Response	Patients, n (%)
Save a copy for my records	98 (32.8)
Share them with my primary care doctor if they don’t have them already	76 (25.4)
Share them with other doctors for a potential second opinion	44 (14.7)
Other (please specify)	31 (10.4)
Share them on social media	9 (3)
None of the above	135 (45.2)

Of the 9 patients who shared their images on social media, their images were posted on Facebook or Instagram, with 2 patients posting on both platforms. In response to asking why they posted their images to social media, the vast majority (8/9, 89%) stated they intended to inform their friends, family, or both. One respondent shared ultrasound images of their pregnancy, and 1 respondent did not answer this open-ended question. Additionally, 45.1% (135/299) of the respondents reported taking no action with their images and 10.4% (31/299) indicated that they did something else with their images but provided no specifics on this. Tables 5 and 6 reveal the breakdown of the electronic devices used by patients to view their images online as well as the technical difficulties experienced, respectively.

**Table 5:** Patient responses to the question, “Which type of device(s) did you use to view your images? Select all that apply” (N=299).^a^

Response	Patients, n (%)
Desktop/laptop computer	172 (57.5)
Smartphone	154 (51.5)
Tablet/iPad	59 (19.7)

^a^The percentage of each device user sums to greater than 100% because each patient was able to select more than one type of viewing device.

**Table 6:** Technical issues expressed by the patients in response to the open-ended statement, “Did you experience any technical issues with viewing your radiology images?” (N=103).

Concern	Patients, n (%)
Trouble viewing (image did not load, froze, server down, etc)	43 (40.2)
Navigation issues	25 (24.3)
Required multiple refreshes/attempts to work	15 (14.6)
Phone-specific issue	7 (6.8)
Browser-specific issue	4 (3.9)
Tablet/iPad-specific issue	3 (2.9)
None reported	6 (5.8)

We had 299 patients respond to the survey, but some patients indicated that they used more than one type of device to view their images, which resulted in 385 data points that included patients who used either just a single device or multiple devices. Approximately 44.6% (172/385) of the patients viewed their images on a desktop or laptop computer, with 22.1% (38/172) of the users reporting technical difficulties. About 40% (154/385) of the respondents viewed their images on their smartphone, with 33.1% (51/154) of the users reporting technical difficulties. Approximately 15.3% (59/385) of the respondents used a tablet to view their images, with 24% (14/59) of the users reporting technical difficulties. The total percentage of computer, smartphone, and tablet users sums to greater than 100% because patients commonly view their images on more than one device and could select more than one type of viewing device in our survey. Lastly, we asked patients a series of open-ended questions to better understand their experiences (ie, questions 6, 11-13, and 15 available in Multimedia Appendix 1). We received 231 open-ended responses regarding the question of perceived benefits of viewing radiology images online. Of these respondents, 41.9% (97/231) stated that online viewing of radiological images increased their understanding of their medical issue and 18.6% (43/231) reported access to the images being beneficial for seeing, saving, and sharing their images with family or their doctor. Patients were also asked about their concerns regarding viewing radiology images online, to which we received 197 open-ended responses; 28 of these respondents (14.2%) stated that online viewing of their radiology images caused them confusion, 14 (7.1%) reported feeling that they had no one with whom they could discuss their questions, and 128 (64.9%) respondents reported no concerns with viewing their actual images. Table 7 illustrates representative patient quotations from open-ended questions 6, 12, and 13. Sample responses to question 6 are listed under “General Reactions,” sample responses to question 12 are listed under “Benefits,” and sample responses to question 13 are listed under “Concerns.”

**Table 7:** Representative patient responses.

Response type	Quote 1 examples	Quote 2 examples
General reaction	…*This is an excellent feature that assists me as a patient in making my healthcare decisions.*	…*The report is more important to me than the images. I really have no idea what I’m looking at with the images.*
Benefits	…*A sense of involvement in my treatment and health. Usually you never get to see your images, so it felt empowering.*	…*I was able to ask my doctor more informed questions when we spoke. As a result, I felt I had fewer unresolved questions later, and worried less.*
Concerns	…*I would like access the report at the same time I get access to the images. Seeing the images without the results is not an effective way to share the information.*	…*I did not have a professional available to explain the results. I had to wait for my appointment with my oncologist.*

## Discussion

### Principal Findings

As anticipated from our prior work, a majority (258/299, 86.3%) of the postintervention respondents had a favorable experience of viewing their radiology images online [10]. Additionally, the majority of the respondents reported finding high levels of value in viewing both their radiology reports and images online. The perceived value patients experienced viewing their reports and radiology images was higher than that anticipated by the responses in our prior survey, perhaps suggesting that patients may underestimate the value of having access to these until they have the opportunity to do so. This highlights the importance of patient accessibility to their personal health information. For example, 1 patient stated, “I like to be informed on what’s going on and this is the best way to do it, where I can see the X-ray or CT scan and then listen to their follow-up on it, which helps me learn about my illness.” Other patients reported feeling “empowered,” while one stated that viewing their images “made me feel more involved/in control of my injury. ‘A picture is worth a thousand words’ comes to mind.”

It has been theorized that patient fears related to confidentiality, lack of awareness of patient portals, and negative experiences when first accessing portals, as well as numerous socioeconomic factors related to education, limited internet access, and being members of certain racial minorities can lead to significant skepticism regarding patient portals and results in reduced use of these tools [11-14]. Although our study is not designed to assess the myriad of factors that may cause patients to be reticent to view their images online, in all categories regarding patient concerns, postimplementation patient concerns were even lower than those anticipated from our prior work [10]. We suspect that the universally lower rates of adverse experiences reported by our respondents in this study compared to what was anticipated could be a result of assumption-making or insufficient preliminary understanding about the proposed implementation. Our postintervention survey by design samples only those patients who have used and are familiar with the portal. Actually using and being familiar with the portal itself and how it functions may allay wariness or other concerns that a patient may have anticipated before actually being able to utilize such functionality. Nevertheless, there remains ample opportunity to address concerns raised by patients, thus improving overall access and patient experience. Potential solutions include better instructional material on how to access these images on the various desktop, tablet, and mobile platforms, the ability to demo the image viewer to allow patients to practice using them and gain familiarity with their use before using them to access their own images in a higher stress environment, and perhaps target these interventions at specific patient groups more likely to experience such issues (the older adults and certain socioeconomic populations).

Over half of the patients (174/299, 58.2%) reported having questions about their images after viewing them. Although most of these patients discussed their questions with their referring doctor, 7.7% (23/299) of the patients did not ask anyone about their questions. Those that did not ask anyone about their questions indicated a myriad of reasons for this, including “not knowing who to ask,” feeling that the “doctor did not have time to answer questions during their visit,” that they felt “embarrassed” or did not want to “bother” anyone, or simply that they decided to “wait to ask questions at their next appointment.” For those patients who may not know where to direct their questions, possible solutions include an in-application chat-style box, question form, and an easily accessible contact list of providers involved in that patient’s care, such as providing contact information of the radiologist at the bottom of every report so that patients can easily contact the radiologist with questions [15].

A significant concern cited by up to 14.2% (28/197) of the respondents was confusion brought upon by viewing the images themselves. Perhaps this confusion is not at all surprising as radiology images are quite complex, and the verbiage used in the dictated reports describing these images are tailored for medical professionals rather than for helping patients understand their images themselves. One possible intervention that may help to reduce this confusion would be to provide reports that are more patient-friendly, either utilizing simplified language that can be understood by patients without medical backgrounds or providing patient-friendly explanations/definitions and diagrams directly within the patient report to allow patients to better understand the reports and their corresponding images [16]. Moreover, some reporting systems allow for hyperlinks within reports that can bring patients to the specific image being discussed, which can help patients correlate between the findings described on the reports and the corresponding images. Additional visual aids such as normal comparisons may be of use and was specifically suggested by 1 respondent who stated “it would be nice to see what a normal image would look like against mine so I can see what’s different.”

Between a quarter and a third of the patients reported technical difficulties with viewing their images online using their electronic device, with the highest incidence rate for those using smartphones. Many patients reported issues with loading and navigating their images. One patient stated that “enlarging or reducing images is difficult. Tools are not user-friendly for nonmedical people.” Another said that image viewing “works more easily on iPad or computer.” One explanation for these experiences is that no substantial modifications were made to the provider image-viewer to aid in functionality when it was adapted for patient use. Specifically, no explanations are provided within the image viewer tool as to what individual buttons mean. Although some tools such as a magnifying glass may be self-explanatory to patients, other tools such as Window/Level buttons etc are far less intuitive and are likely to easily confuse patients. Additionally, the image viewer tool is optimized for desktop/browser-based interactions, but a significant proportion of our patients accessed these images via touch screen mobile devices, which make interaction with the image-viewer tool more difficult. A number of potential solutions for these problems should be considered. An in-application help feature explaining the function of buttons within the tool may be of benefit to improve the experience. Additional optimization of image viewing tools for mobile devices/tablets will also make the image viewing experience far easier. Health care systems should continue investing in their information technology infrastructure and upgrading image-viewing platforms to meet the needs of their patients.

It is notable that patients in our study reported significant lower rates of saving copies of images for their records (98/299, 32.8%), sharing their images with their primary care physician (76/299, 25.4%), or sharing their images with other providers (44/299, 14.7%) than anticipated from our prior work. In fact, 45.2% (135/299) of our survey respondents reported doing nothing with their images, as opposed to only 5.7% of anticipating the same in our prior work [10]. Although this could be due to a multitude of different factors, a significant factor is likely that in our image viewer, no modifications were made to increase the ease of saving and sharing images from their device. Additionally, technical difficulties experienced by patients, especially on mobile devices, likely made such image sharing far more difficult than originally anticipated.

A small percentage of the patients (9/299, 3%) posted their radiology images to social media platforms, that is, Facebook and Instagram. Patients who engaged with social media did so primarily to inform and reassure their friends and family. One patient reported sharing their images “for my family only to keep them better informed, to answer their questions and to reassure them.” Another patient expressly used the platforms to share pregnancy ultrasound images: “Seeing prenatal ultrasounds was fun for family members who couldn’t be at appointments because of COVID. I also love reviewing the images to see our baby grow.”

### Limitations

We have refrained from a detailed direct comparison of the results of this study survey to our work prior to the implementation of the image viewer portal owing to differences in the populations sampled. Our prior work sampled members of the UCHealth Insights community, many of whom are patients at UCHealth, but includes other health care decision makers in the community as well. Patients were a sample limited to MHC portal users, as this is the only way to survey patients who had viewed their radiology images. As such, only general comparisons to prior work are discussed here. Although our survey demonstrated a largely favorable experience with viewing their images online, the literature on patient satisfaction surveys does show that the most satisfied patients are more likely to participate in surveys than the dissatisfied, and this tendency produces a positive bias in favorability scores. Additionally, the response rate for our survey is 16.4% (299/1825), which while within the expected range for email survey responses based on previous work in our health system and within the lower end of range of reported response rates in the literature still leads to the possibility that dissatisfied patients may have not responded, and we cannot infer bias or motivation to reply in our analysis [17,18]. Our respondents come from a single center, and the age/sex of the respondents (online image viewers) do not perfectly reflect the age/sex of the patients in our health system in general. Our surveyed patients were selected by sampling those who had most recently accessed their images online over a 5-day period of the study. Given the anonymous nature of the survey, we are unable to consider the severity of patient illness or the cognitive impairment of the survey respondents and cannot assess how these factors may impact survey results. Lastly, as a quality improvement project, our findings are not intended to be generalizable.

### Future Directions

As more health care systems move to making online viewing of radiology images available to their patients, our understanding of the associated benefits and risks will continue to grow. Although our respondents largely indicated favorable experiences with viewing their radiology images on MHC, future investigations should determine the reproducibility of our findings in more diverse population groups. A small but significant set of concerns were raised in terms of patient anxiety and worries caused by viewing radiology images before seeing the associated report or being able to discuss the results with a health care provider. Institutions that offer patients the ability to view their radiology images online should consider ways of mitigating these concerns during the implementation of these novel solutions. We also noticed a lower rate of utilization of images (saving copies for their records, sharing with primary care physician and other providers) than anticipated. Further insight into this surprising result is warranted, as it highlights a potential target for improvement.

Additionally, future investigations focusing on user interface, technical improvements, and additional features desired by patients could help to improve the overall patient experience interacting with their web-based portal and image-viewing platform. Although our survey focused on the patient perspective, we believe that a comprehensive understanding of the impact of online radiology image viewing requires the consideration of the provider perspective as well. In the experience of our radiology leaders and clinician leaders, surprisingly few concerns have been raised in the past 2 years. By seeking out both patient and provider attitudes, we would be able to optimize the experience for both.

### Conclusions

To our knowledge, this is the first report to describe patients’ experience in viewing their radiology images through a web-based patient portal. Patients expressed a high level of satisfaction and a low incidence of negative experiences. Patients who viewed their radiology images reported increasing trust, autonomy, reassurance, and medical understanding, with few expressing concerns such as anxiety or confusion. From these results, we see opportunities to further improve the patient experience with online image viewing. Overall, our experience and that of our patients has been positive with rare concerns. We suggest that patient access to radiology images, such as patient access to radiology reports, is highly desired by patients and is operationally practical. Other health care institutions should consider offering radiology images online in the pursuit of information transparency.

## Appendix

Multimedia Appendix 1 Postintervention survey design that was emailed to our target population.

## Abbreviations

MHC: My Health Connection
