# Supplementary material for: Understanding Patient Experiences, Opinions, and Actions Taken After Viewing Their Own Radiology Images Online: Web-Based Survey
Source: JMIR Form Res. 2022 Apr 25;6(4):e29496. doi: 10.2196/29496 (PMC9086874; doi:10.2196/29496)
Supplement: Multimedia Appendix 1 [file formative_v6i4e29496_app1.docx]

**Survey Design:**

*Thank you for taking the time to provide your feedback. This survey will take about five minutes of your time. It includes questions about radiology reports and images.*

1. Our records indicate that you recently had radiology images (X-ray, CT, MRI, ultrasound) at UCHealth. Is this correct?
   1. Yes
   2. No
2. Which of the following do you recall viewing within MyHealthConnection (your online patient portal where you are able to view your test results, refill prescriptions, etc.)? Select all that apply.
   1. Radiology reports (describe the findings in the radiology images)
   2. Radiology image(s)
   3. Neither
3. When were the radiology images taken?
   1. As a patient admitted to the hospital
   2. As part of a clinic or radiology appointment
   3. Other/not sure
4. Which type of device(s) did you use to view your images? Select all that apply.
   1. Desktop/laptop computer
   2. Tablet/IPad
   3. Smartphone
5. Did you experience any technical issues with viewing your radiology images?
   1. No
   2. Yes (please explain)
6. What did you think about viewing your images online? (open end)
7. Please rate the value of viewing each of the following within your online patient portal: (1 to 5 scale)
   1. Radiology report (describe the findings in the radiology images)
   2. Radiology images
8. Please rate the following statements on a scale from 1 to 5 where 1 is Strongly Disagree and 5 is Strongly Agree.

“Viewing my radiology images online caused me to…

(randomize order)

- 1. …worry more.”
  2. …feel confused/have a lot of questions.”
  3. …feel more in control
  4. …better understand my medical condition.”
  5. …feel reassured.”
  6. …better follow recommendations.”
  7. …trust my doctors more.”
  8. …find errors in my radiology reports.”

1. Which of the following have you done with your radiology images? Select all that apply.
   1. Share them with my primary care doctor if they don’t have them already
   2. Share them with other doctors for a potential second opinion
   3. Share them on social media
   4. Save a copy for my records
   5. Other (please specify)
   6. None of the above
2. (If 9c) On which social media platform(s) did you share your radiology image(s)?
   1. Facebook
   2. Instagram
   3. Snapchat
   4. Reddit
   5. TikTok
   6. Twitter
   7. Pinterest
   8. Other
3. Why did you share your radiology image(s) on social media? (open end)
4. What were the benefits of viewing your radiology images online? (open end)
5. Please explain any concerns about viewing your radiology images online. (open end)
6. If you had questions about your images after viewing them online, who did you discuss them with? Select all that apply.
   1. The doctor who referred me to radiology
   2. The radiologist who wrote my radiology report
   3. Other (please specify)
   4. I had questions but did not ask anyone
   5. I did not have questions
7. (If #14=d) Why didn’t you discuss your questions with anyone? (open end)
8. Overall, how was your experience viewing your radiology images in MyHealthConnection?
   1. 1 to 5 scale

The following questions are for classification purposes only.

1. What is your gender?
   1. Male
   2. Female
   3. Other non-binary
   4. Prefer not to say
2. What is your age?
   1. 18-24
   2. 25-34
   3. 35-44
   4. 45-54
   5. 55-64
   6. 65+
3. What is your annual household income?
   1. Less than $25k
   2. $25k - $49k
   3. $50k - $74k
   4. $75k - $99k
   5. $100k - $149k
   6. $150k- $199k
   7. $200,000+
   8. Prefer not to say
4. What is the level of schooling that you have completed?
   1. I did not graduate high school
   2. High school graduate, diploma or the equivalent (for example: GED)
   3. Some college
   4. Associates degree
   5. Bachelor’s degree
   6. Master’s degree
   7. Professional degree.
   8. Doctorate degree.

*Thanks again for your time in completing this survey!*
